# Role of STAT3 dependent SOX2 and CD24 expression in melanoma cell adaptive resistance towards targeted therapies

**DOI:** 10.18632/oncotarget.26718

**Published:** 2019-03-01

**Authors:** Laura Hüser, Peter Altevogt, Jochen Utikal

**Affiliations:** Skin Cancer Unit, German Cancer Research Center, Heidelberg, Germany; Department of Dermatology, Venereology and Allergology, University Medical Center Mannheim, Ruprecht-Karl University of Heidelberg, Mannheim, Germany

**Keywords:** melanoma, targeted therapy, resistance, Sox2, BRAF

Melanoma, the deadliest form of skin cancer, is often characterized by mutations of genes involved in the MAP Kinase signaling pathway leading to a hyperactivation of the pathway and thereby uncontrolled cellular proliferation and survival of the malignant cells. In recent times drugs specifically targeting the MAP Kinase signaling pathway were therefore developed. These so called "targeted therapies" like BRAF or MEK inhibitors are very powerful in the beginning of treatment but are limited due to the occurrence of resistance [1]. Therefore, it is of great importance to find modalities that can counteract the resistance and allow prolonged "targeted therapy". To this end, it is essential to understand the molecular mechanisms of tumor cell response to the treatment.

The Signal transducer and activator of transcription 3 (STAT3) was shown to play a central role in resistance towards targeted therapies [2]. Moreover, STAT3 was demonstrated to be upregulated in cancer stem cells [3] as well as together with SOX2 in clustered circulating tumor cells, which have a high metastatic potential [4]. In our publication [5] we could demonstrate that the initial STAT3 activation induced by BRAF inhibitor treatment resulted in an increased expression of SOX2 and CD24 which were both associated to an increased resistance since overexpression of either SOX2 or CD24 resulted in a significantly higher tolerance against BRAF inhibitors. In contrast, the knock down of both molecules rendered cells more sensitive towards the treatment.

SOX2 was demonstrated before to be a cancer stem cell marker and its expression is increased in melanospheres which showed a higher resistance towards the BRAF inhibitor vemurafenib [6, 7]. Interestingly, we could show that SOX2 is able to bind to the CD24 promotor and thereby promoting the CD24 expression. This result established a link between SOX2 and CD24 expression. In other cancers CD24 was shown to be involved in tumor cell proliferation, adhesion, migration and invasion [8]. One way to explain how CD24 as a GPI-anchored membrane protein can regulate these cellular features is by promoting Src and STAT3 signaling [9]. Indeed, it was demonstrated that CD24 is an important organizer of lipid rafts which are signaling domains at the plasma membrane. Thus, Src and STAT3 signaling is enhanced in cells where CD24 is expressed. In response to BRAF inhibitor treatment, melanoma cells upregulate STAT3 activity resulting in higher expression of SOX2. SOX2 in turn promotes the expression of CD24 finally resulting in an increased Src and STAT3 activity. We speculate that this is most likely due to a CD24 dependent change in the compositions of lipid rafts similar as described for other cancers [9]. But it should be borne in mind that SOX2 is not the only factor that can augment CD24 expression. For example, in colon cancer CD24 expression was shown to be controlled by COX2 and PGE2 synthesis, which is directly regulated by b-catenin [10].

**Figure 1:**
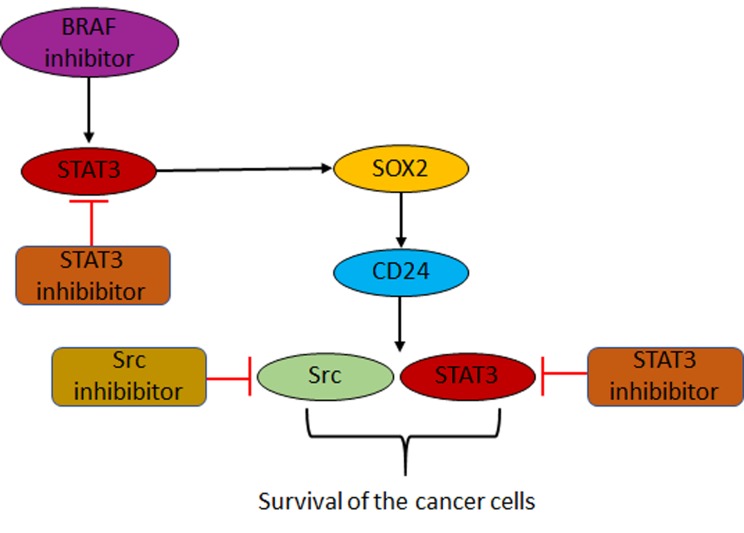
Novel mechanism of adaptive resistance towards BRAF inhibitors in melanoma BRAF inhibitor treatment leads to an increased STAT3 activation. STAT3 promotes the expression of SOX2 and SOX2 then induces the expression of CD24. CD24 in turn promotes Src and STAT3 activity most likely due to a change in the lipid raft composition favoring cancer cell survival. Thus, inhibitors targeting STAT3 or Src can help to overcome this mechanism of adaptive resistance.

It appears that for melanoma cells CD24 upregulation constitutes an escape mechanism by which the cells survive the initial and toxic exposure to the BRAF inhibitor. The surviving cells are then able to acquire additional long-term mechanisms of drug resistance. Our results suggest that this escape mechanism can be blocked by using Src or STAT3 inhibitors. Hence, the use of these inhibitors even in the more resistant SOX2 and CD24 overexpressing cells lead to a higher sensitivity towards the BRAF inhibitor treatment [5]. STAT3 plays a very crucial role as it is important in the initial increase of SOX2 and CD24 expression. In addition, STAT3 at the end is higher activated due to the increased CD24 level and therefore might be the perfect target to increase the efficacy of "targeted therapy". The mechanism of adaptive resistance found within our work is summarized in Figure 1.

This mechanism of adaptive resistance in melanoma cells helps to understand how some of the cancer cells manage to escape the cytotoxic activity of BRAF inhibitors and are able to acquire long-term mechanisms of resistance resulting in cancer relapse in the patients. Thus, we highly recommend to proof if the use of Src and STAT3 inhibitors can help patients to delay the onset of resistance or in best case prevent resistance towards BRAF inhibitors.
